# Factors affecting drug retention of Janus kinase inhibitors in patients with rheumatoid arthritis: the ANSWER cohort study

**DOI:** 10.1038/s41598-021-04075-0

**Published:** 2022-01-07

**Authors:** Kosuke Ebina, Toru Hirano, Yuichi Maeda, Wataru Yamamoto, Motomu Hashimoto, Koichi Murata, Akira Onishi, Sadao Jinno, Ryota Hara, Yonsu Son, Hideki Amuro, Tohru Takeuchi, Ayaka Yoshikawa, Masaki Katayama, Keiichi Yamamoto, Yasutaka Okita, Makoto Hirao, Yuki Etani, Atsushi Kumanogoh, Seiji Okada, Ken Nakata

**Affiliations:** 1 Department of Musculoskeletal Regenerative Medicine, Osaka University Graduate School of Medicine, Osaka, Japan; 2 Department of Rheumatology, Nishinomiya Municipal Hospital, Hyogo, Japan; 3 Department of Respiratory Medicine and Clinical Immunology, Osaka University Graduate School of Medicine, Osaka, Japan; 4 Integrated Frontier Research for Medical Science Division, Institute for Open and Transdisciplinary Research Initiatives, Osaka University, Osaka, Japan; 5 Department of Health Information Management, Kurashiki Sweet Hospital, Okayama, Japan; 6 Department of Advanced Medicine for Rheumatic Diseases, Graduate School of Medicine, Kyoto University, Kyoto, Japan; 7 Department of Clinical Immunology, Osaka City University Graduate School of Medicine, Osaka, Japan; 8 Department of Rheumatology and Clinical Immunology, Kobe University Graduate School of Medicine, Hyogo, Japan; 9 Department of Orthopaedic Surgery, Nara Medical University, Nara, Japan; 10 First Department of Internal Medicine, Kansai Medical University, Osaka, Japan; 11 Department of Internal Medicine (IV), Osaka Medical and Pharmaceutical University, Osaka, Japan; 12 Department of Rheumatology, Osaka Red Cross Hospital, Osaka, Japan; 13 Information Technology Center, Wakayama Medical University, Wakayama, Japan; 14 Department of Orthopaedic Surgery, Osaka University Graduate School of Medicine, Osaka, Japan; 15 Department of Health and Sport Sciences, Osaka University Graduate School of Medicine, Osaka, Japan

**Keywords:** Rheumatic diseases, Outcomes research

## Abstract

This multi-center, retrospective study aimed to clarify the factors affecting drug retention of the Janus kinase inhibitors (JAKi) including baricitinib (BAR) and tofacitinib (TOF) in patients with RA. Patients were as follows; females, 80.6%; age, 60.5 years; DAS28-ESR, 4.3; treated with either BAR (n = 166) or TOF (n = 185); bDMARDs- or JAKi-switched cases (76.6%). The reasons for drug discontinuation were classified into four major categories. The drug retention was evaluated at 24 months using the Kaplan–Meier method and multivariate Cox proportional hazards modelling adjusted by confounders. Discontinuation rates for the corresponding reasons were as follows; ineffectiveness (22.3%), toxic adverse events (13.3%), non-toxic reasons (7.2%) and remission (0.0%). Prior history of anti-interleukin-6 receptor antibody (aIL-6R) ineffectiveness significantly increased the risk of treatment discontinuation due to ineffectiveness (p = 0.020). Aging (≥ 75 years) (p = 0.028), usage of PSL ≥ 5 mg/day (p = 0.017) and female sex (p = 0.041) significantly increased the risk of treatment discontinuation due to toxic adverse events. Factors not associated with treatment discontinuation were: number of prior bDMARDs or JAKi, concomitant MTX usage, difference of JAKi, and prior use of TNF inhibitor, CTLA4-Ig or other JAKi.

## Introduction

The recommendations of the 2019 European League Against Rheumatism (EULAR) stated that the efficacies of anti-interleukin (IL)-6 receptor antibody (aIL-6R; tocilizumab and sarilumab), cytotoxic T lymphocyte-associated antigen-4-Ig (CTLA4-Ig; abatacept) and Janus kinase inhibitors (JAKi) such as baricitinib (BAR; a JAK1 and JAK2 inhibitor) and tofacitinib (TOF; a JAK1 and JAK3 inhibitor) are considered equivalent to those of tumor necrosis factor inhibitors (TNFi) in both Phase II and Phase III treatments of rheumatoid arthritis (RA)^1^. The authors reported no significant differences in outcomes between biological disease-modifying antirheumatic drugs (bDMARDs) and JAKi therapy, irrespective of their targets.

JAKi inhibits the JAK-signal transducer and activator of transcription pathways system, which leads to the inhibition of IL-6 and various other cytokines^2^. Five JAKi, including TOF (2013), BAR (2017), peficitinib (2019), upadacitinib (2020) and filgotinib (2020), have been approved for use in Japan—the only country to have approved five JAKi.

In real-world settings, JAKi tends to be introduced in patients with intolerance to methotrexate (MTX) due to comorbidities or with multiple bDMARDs failures—quite different from those recruited in randomised controlled trials. Therefore, it is of great interest to investigate factors affecting the effectiveness and safety of JAKi in ‘difficult-to-treat’ RA patients, especially those who were previously treated with TNFi, aIL-6R, CTLA4-Ig or another JAKi.

The performance of bDMARDs has increasingly been investigated through recent cohort-based observational studies^3,4^ in which drug retention is considered a major index of both treatment safety and effectiveness^5,6^. We have recently reported the drug retention rates of bDMARDs^7–12^, factors affecting the efficacy of bDMARDs^13,14^ and factors affecting the achievement of bDMARDs-free remission^15^ on the basis of findings from our cohort. The aim of the present multicenter, retrospective study is to clarify the factors affecting drug retention of a JAKi (BAR or TOF) in real-world settings.

## Methods

### Study design and patients

The Kansai Consortium for *We*ll-being of *R*heumatic Disease Patients (*ANSWER*) cohort is an observational, multicenter registry of patients with RA in the Kansai district of Japan^7–12^. Data were retrospectively collected from patients who were examined at seven major university-related hospitals (Kyoto University, Osaka University, Osaka Medical College, Kansai Medical University, Kobe University, Nara Medial University and Osaka Red Cross Hospital). RA was diagnosed on the basis of the 1987 RA classification criteria of the American College of Rheumatology (ACR)^16^ or the 2010 ACR/EULAR RA classification criteria^17^.

Patients who were treated with either BAR or TOF between 2013 and 2020, with complete data on the start and discontinuation dates and the reasons for discontinuation, were included in this study. Additional data were collected, including baseline demographic data (age, sex); disease duration; disease activity (disease activity score in 28 joints using erythrocyte sedimentation rate [DAS28-ESR]); Clinical Disease Activity Index (CDAI) score; concomitant doses (calculated as a blank when not combined) and ratios of MTX and glucocorticoid (GC) (prednisolone [PSL] equivalent); concomitant ratios of other conventional disease-modifying antirheumatic drugs (csDMARDs), such as salazosulfapyridine, bucillamine, iguratimod, tacrolimus and leflunomide; rheumatoid factor (RF) and anti-cyclic citrullinated peptide antibody positivity; and Health Assessment Questionnaire Disability Index score^7–9^. Patients were categorized by age: young, < 65 years; old, 65–74 years; and very old, ≥ 75 years^18^ and by concomitant dose of PSL (< 5 or ≥ 5 mg/day)^19^ because previous reports had demonstrated that these categories are associated with drug retention of bDMARDs and JAKi.

In Japan, public national health insurance covers 70%–90% of medical expense, and bDMARDs or JAKi can be administered at the discretion of attending rheumatologists, in accordance with the Japan College of Rheumatology guidelines^20–22^. The dose of each agent is determined in accordance with the manufacturer’s recommendation. Drug retention was retrospectively evaluated as the duration until definitive treatment interruption. The reasons for discontinuation were classified into four major categories as follows: (1) lack of effectiveness (including primary and secondary); (2) toxic adverse events (infection, skin or systemic reaction and other toxic events, including haematologic, pulmonary, renal, cardiovascular complications and malignancies); (3) non-toxic reasons (patient preference, change in hospital, desire for pregnancy, etc.); and (4) disease remission^7–9,11,12^. Physicians were allowed to cite only one reason for discontinuation.

### Statistical analyses

Differences in baseline clinical characteristics between the groups were assessed using the Mann–Whitney U test (for continuous variables) and the chi-squared test (for categorical variables). The Kaplan–Meier method adjusted by potential confounders was used to examine the survival curves for the agents, as determined by the specified causes. Hazard ratios (HR) and Cox p-value for each discontinuation reason of treatment at 24 months were analyzed using multivariate Cox proportional hazards modelling, by including all of the potential confounders and excluding other non-relevant discontinuation reasons^3,7–9^. The analysis was adjusted for potential confounders that could influence drug retention, as previously described (age; sex; disease duration; concomitant PSL and MTX use; difference of JAKi; number of switched bDMARDs or JAKi; prior use of TNFi, aIL-6R, CTLA4-Ig or other JAKi)^3,23–26^. Some minor missing baseline data such as disease activities were extracted by last observation carried forward, which were excluded from the adjustment confounders. In evaluating the effects of prior treatment on drug retention, patients with at least one history of discontinuation due to ineffectiveness in the same drug categories (TNFi, aIL-6R, CTLA4-Ig or JAKi) were categorized as “drug ineffectiveness”. Other 3 reasons for discontinuation excluding ineffectiveness was categorized as “drug intolerance”. Statistical analyses were performed using EZR (Saitama Medical Center, Jichi Medical University, Saitama, Japan), which is a graphical user interface for R software (R Foundation for Statistical Computing, Vienna, Austria)^27^. A two-sided p value < 0.05 was considered statistically significant.

### Ethical statement

The representative facility of this registry was Kyoto University, and this observational study was conducted in accordance with the Declaration of Helsinki, with the approval of the ethics committees of the following seven institutes: Kyoto University (2016-03-24/approval No. R053), Osaka University (2015-11-04/approval No. 15300), Osaka Medical College (2014-07-14/approval No. 1529), Kansai Medical University (2017-11-21/approval No. 2014625), Kobe University (2015-03-20/approval No. 1738), Nara Medial University (2018-01-23/approval No. 1692), and Osaka Red Cross Hospital (2015-09-01/approval No. 644). The board of the Osaka University Hospital Ethics Committee waived the requirement for patient informed consent because of the anonymous nature of the data. Written informed consent was obtained from the participants in other institutes.

## Results

### Clinical characteristics

Table 1 presents the baseline clinical characteristics of the patients at initiation of treatment with each agent. Most of the patients who experienced prior JAKi were switched from TOF to BAR (n = 30) or from BAR to TOF (n = 8). Overall, patients were treated by a low dose and ratio of MTX, and had mostly switched from other bDMARDs or JAKi, suggesting ‘difficult-to-treat’ backgrounds.

**Table 1:** Patients’ clinical characteristics at initiation of treatment with each agent.

Variable	BAR (n = 166)	TOF (n = 185)	p value
Age (years)	60.2 ± 13.5	60.7 ± 13.1	0.87
Female sex (%)	86.7	75.1	0.009
Disease duration (years)	12.6 ± 10.6	9.7 ± 8.3	0.016
RF positivity (%)	86.1	81.6	0.50
ACPA positivity (%)	82.0	83.1	0.93
DAS28-ESR	4.3 ± 1.3	4.3 ± 1.3	0.98
CDAI	17.2 ± 11.0	18.8 ± 11.1	0.16
HAQ-DI	0.9 ± 0.7	0.9 ± 0.8	0.81
PSL use (%)	42.8	50.3	0.19
PSL dose (mg/day)	4.7 ± 3.2	5.7 ± 3.3	0.022
MTX use (%)	64.5	57.3	0.048
MTX dose (mg/week)	8.7 ± 3.1	9.2 ± 3.3	0.35
SASP use (%)	11.4	23.8	0.004
BUC use (%)	7.8	8.6	0.93
IGU use (%)	13.3	17.8	0.30
TAC use (%)	15.7	9.7	0.13
LEF use (%)	0.0	0.0	N.A
bDMARDs or JAKi naive (%)	22.3	24.3	0.75
2nd bDMARDs or JAKi (%)	23.5	24.3	0.86
3rd bDMARDs or JAKi (%)	26.5	16.2	0.018
≥ 4th bDMARDs or JAKi (%)	27.7	35.1	0.14
Prior TNFi use (%)	57.8	65.9	0.15
Prior aIL-6R use (%)	36.1	40.5	0.46
Prior CTLA4-Ig use (%)	31.9	25.4	0.22
Prior JAKi use (%)	20.5	6.5	< 0.001
Prior JAKi	TOF (n = 30), BAR (n = 1), PEF (n = 3)	TOF (n = 4), BAR (n = 8)	N.A

Values are presented as mean ± standard deviation or percentage. Differences between the groups were assessed by the Mann–Whitney U test or the chi-squared test.

*N.A.* not applicable, *BAR* baricitinib, *TOF* tofacitinib, *RF* rheumatoid factor, *ACPA* anticyclic citrullinated peptide antibody, *DAS28-ESR* Disease Activity Score in 28 joints using erythrocyte sedimentation rate, *CDAI* clinical disease activity index, *HAQ-DI* Health Assessment Questionnaire disability index, *PSL* prednisolone, *MTX* methotrexate, *SASP* salazosulfapyridine, *BUC* bucillamine, *IGU* iguratimod, *TAC* tacrolimus, *LEF* leflunomide, *bDMARDs* biological disease-modifying antirheumatic drugs, *JAKi* Janus kinase inhibitor, *TNFi* tumour necrosis factor inhibitors, *aIL-6R* anti-interleukin-6 receptor, *CTLA4-Ig* cytotoxic T lymphocyte-associated antigen-4-Ig, *PEF* peficitinib.

### Reasons and rates of drug discontinuation

In total, the adjusted discontinuation rates for the corresponding reasons at 24 months were as follows: lack of effectiveness (22.3%), toxic adverse events (13.3%), non-toxic reasons (7.2%) and remission (0.0%). We further investigated the factors affecting discontinuation due to lack of effectiveness and toxic adverse events, using multivariate Cox proportional hazards modelling. Prior use of aIL-6R significantly increased the risk of treatment discontinuation due to lack of effectiveness (HR, 2.07; p = 0.021), and prior use of TNFi tended to increase the risk of treatment discontinuation due to lack of effectiveness (HR, 1.90; p = 0.075) (Table 2).

**Table 2:** Cox proportional hazard analysis for the risk factors of treatment discontinuation due to lack of effectiveness.

Variable	HR (95% CI)	p value
Prior aIL-6R use (%)	2.07 (1.12–3.83)	0.021
Prior TNFi use (%)	1.90 (0.94–3.84)	0.075
Age (years)	0.99 (0.97–1.01)	0.14
Sex (male)	0.65 (0.34–1.21)	0.17
Disease duration (years)	0.98 (0.95–1.01)	0.20
MTX use (%)	1.21 (0.71–2.04)	0.49
PSL use (≥ 5 mg/day)	0.86 (0.50–1.47)	0.57
Switched number of bDMARDs or JAKi	0.93 (0.71–1.22)	0.58
Difference of JAKi (TOF use)	1.11 (0.64–1.94)	0.70
Prior JAKi use (%)	1.13 (0.48–2.69)	0.78
Prior CTLA4-Ig use (%)	1.02 (0.53–1.96)	0.97

*HR* hazard ratio, *CI* confidence interval, *aIL-6R* anti-interleukin-6 receptor, *TNFi* tumour necrosis factor inhibitors, *MTX* methotrexate, *PSL* prednisolone, *bDMARDs* biological disease-modifying antirheumatic drugs, *JAKi* Janus kinase inhibito, *TOF* tofacitinib, *CTLA4-Ig* cytotoxic T lymphocyte-associated antigen-4-Ig.

As for discontinuation due to toxic adverse events, significant confounders were aging (HR, 1.04; p = 0.015), PSL usage ≥ 5 mg/day (HR, 2.21; p = 0.017) and male sex (HR, 0.33; p = 0.041) (Table 3).

**Table 3:** Cox proportional hazard analysis for the risk factors of treatment discontinuation due to toxic adverse events.

Variable	HR (95% CI)	p value
Age (years)	1.04 (1.01–1.06)	0.015
PSL use (≥ 5 mg/day)	2.21 (1.15–4.23)	0.017
Sex (male)	0.33 (0.11–0.95)	0.041
Disease duration (years)	1.01 (0.98–1.04)	0.64
Prior aIL-6R use (%)	0.83 (0.35–1.97)	0.67
MTX use (%)	1.14 (0.58–2.27)	0.70
Prior JAKi use (%)	1.22 (0.41–3.61)	0.72
Difference of JAKi (TOF use)	0.91 (0.45–1.85)	0.79
Prior CTLA4-Ig use (%)	0.96 (0.42–2.19)	0.93
Switched number of bDMARDs or JAKi	1.01 (0.71–1.46)	0.94
Prior TNFi use (%)	1.03 (0.41–2.60)	0.96

*HR* hazard ratio, *CI* confidence interval, *PSL* prednisolone, *aIL-6R* anti-interleukin-6 receptor, *MTX* methotrexate, *JAKi* Janus kinase inhibitor, *TOF* tofacitinib, *CTLA4-Ig* cytotoxic T lymphocyte-associated antigen-4-Ig, *bDMARDs* biological disease-modifying antirheumatic drugs, *TNFi* tumour necrosis factor inhibitors.

The adjusted drug retention rates for the corresponding reasons and the statistical differences between the groups were as follows.

Between BAR and TOF, there were no significant differences in the retention rates due to lack of effectiveness (BAR, 84.6% vs. TOF, 75.9%; p = 0.70) or toxic adverse events (BAR, 82.7% vs. TOF, 87.5%; p = 0.79; data not shown).

**Figure 1:**
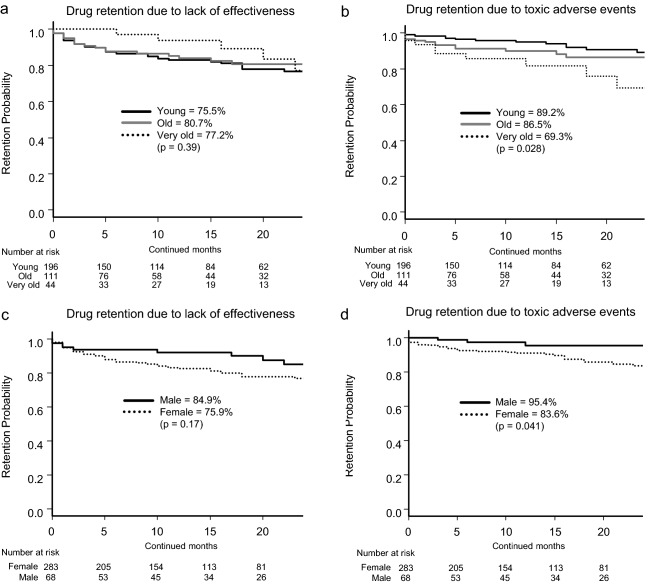
Adjusted drug retention by age and sex. Adjusted drug retention between young (< 65 years), aged (65–74 years), and very old (≥ 75 years) groups, due to (**a**) lack of effectiveness and (**b**) toxic adverse events, and adjusted drug retention between male and female, due to (**c**) lack of effectiveness and (**d**) toxic adverse events.

With regard to age, retention rates due to lack of effectiveness were: young, 75.5% vs. old, 80.7% vs. very old, 77.2% (p = 0.39; Fig. 1a) and those of toxic adverse events were: young, 89.2% vs. old, 86.5% vs. very old, 69.3% (p = 0.028; Fig. 1b). With regard to sex, retention rates due to lack of effectiveness were: male, 84.9% vs. female, 75.9% (p = 0.17; Fig. 1c) and those of toxic adverse events were: male, 95.4% vs. female, 83.6% (p = 0.041; Fig. 1d). Taken together, very old patients (≥ 75 years) and female patients showed higher risk of treatment discontinuation due to toxic adverse events.

**Figure 2:**
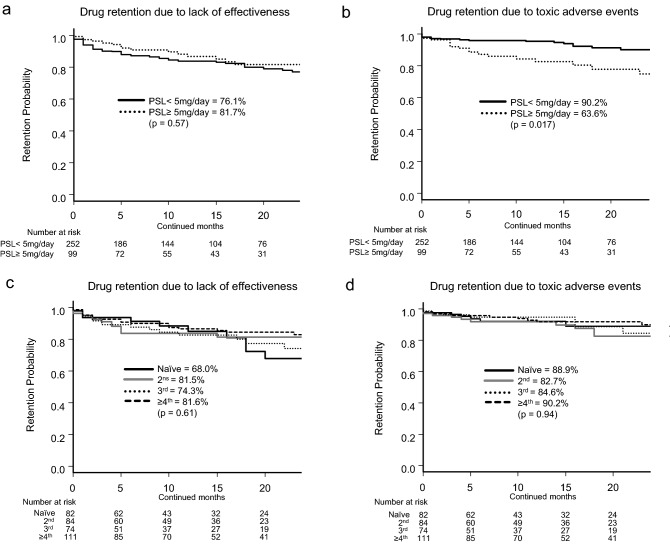
Adjusted drug retention by PSL dose and bDMARD/JAKi therapy. Adjusted drug retention between concomitant PSL < 5 mg/day and PSL ≥ 5 mg/day, due to (**a**) lack of effectiveness and (**b**) toxic adverse events, and adjusted drug retention between switched bDMARD/JAKi groups due to (**c**) lack of effectiveness and (**d**) toxic adverse events. *PSL* prednisolone, *bDMARD* biological disease-modifying antirheumatic drug, *JAKi* Janus kinase inhibitor.

On the other hand, regarding concomitant GC dose, retention rates for discontinuation due to lack of effectiveness were PSL < 5 mg/day, 76.1% vs. PSL ≥ 5 mg/day, 81.7% (p = 0.57; Fig. 2a) and those for toxic adverse events were PSL < 5 mg/day, 90.2% vs. PSL ≥ 5 mg/day, 63.6% (p = 0.017; Fig. 2b). As for the influence of switched number of bDMARDs or JAKi, retention rates due to lack of effectiveness were: naïve, 68.0% vs. 2nd, 81.5% vs. 3rd, 74.3% vs. 4th or more, 81.6% (p = 0.61; Fig. 2c), and those of toxic adverse events were: naïve, 88.9% vs. 2nd, 82.7% vs. 3rd, 84.6% vs. 4th or more, 90.2% (p = 0.94; Fig. 2d). Concomitant PSL ≥ 5 mg/day showed higher risk of treatment discontinuation due to toxic adverse events.

**Figure 3:**
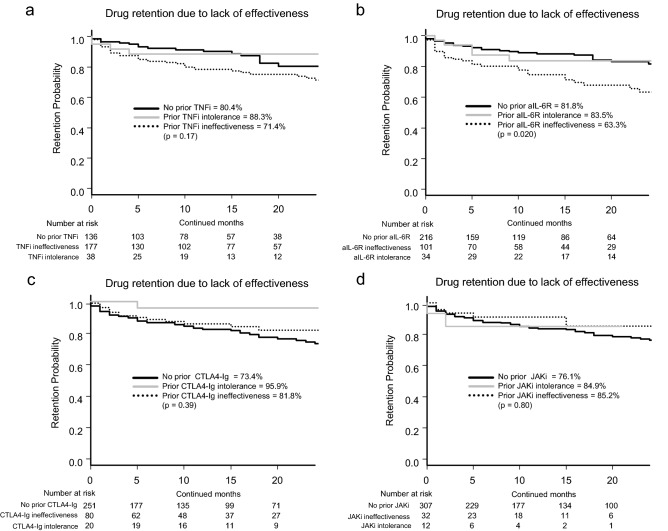
Adjusted drug retention by TNFi, aIL-6R, CTLA4-Ig and JAKi experience. Adjusted drug retention due to lack of effectiveness between (**a**) non-TNFi-experienced, prior TNFi intolerance, and prior TNFi ineffectiveness groups, (**b**) non-aIL-6R-experienced, prior aIL-6R intolerance, and prior aIL-6R ineffectiveness groups, (**c**) non-CTLA4-Ig-experienced, prior CTLA4-Ig intolerance, and prior CTLA4-Ig ineffectiveness groups, (**d**) and non-JAKi-experienced, prior JAKi intolerance, and prior JAKi ineffectiveness groups. *TNFi* tumour necrosis factor inhibitor, *aIL-6R* anti-interleukin-6 receptor, *CTLA4-Ig* cytotoxic T lymphocyte-associated antigen-4-Ig, *JAKi* Janus kinase inhibitor.

Regarding prior TNFi treatment, the retention rates were: without prior TNFi, 80.4% vs. prior TNFi intolerance, 88.3% vs. prior TNFi ineffectiveness, 71.4% (p = 0.17; Fig. 3a). As for prior aIL-6R treatment, the adjusted retention rates were: without prior aIL-6R, 81.8% vs. prior aIL-6R intolerance, 83.5% vs. prior aIL-6R ineffectiveness, 63.3% (p = 0.020; Fig. 3b). Regarding prior CTLA4-Ig treatment, the adjusted retention rates were: without prior CTLA4-Ig, 73.4% vs. prior CTLA4-Ig intolerance, 95.9% vs. prior CTLA4-Ig ineffectiveness, 81.8% (p = 0.39; Fig. 3c). As for prior JAKi treatment, the adjusted retention rates were: without prior JAKi, 76.1% vs. prior JAKi intolerance, 84.9% vs. prior JAKi ineffectiveness, 85.2% (p = 0.80; Fig. 3d). Taken together, history of prior aIL-6R ineffectiveness showed higher risk of treatment discontinuation due to lack of effectiveness. History of prior TNFi, CTLA4-Ig or other JAKi ineffectiveness did not significantly affect following JAKi treatment retention.

## Discussion

Regarding the difference between BAR and TOF, a previous meta-analysis revealed that, in patients with inadequate response to csDMARDs or bDMARDs, BAR and TOF were similarly efficacious^28^, which accords with the results of the present study.

Concerning the effect of aging, the RA-BUILD and RA-BEAM studies of BAR showed similar clinical efficacy between young (< 50 years) and old (≥ 65 years) patients^29^, similar to the phase III and long-term extension studies of TOF^30^. However, elderly patients (≥ 65 years) tended to show a higher rate of discontinuation of BAR treatment due to adverse events (8.8%) than younger patients (< 50 years, 2.3%)^29^, similar to results for TOF^30^. Greater age was associated with increased risk of herpes zoster (HZ)^31^, major adverse cardiovascular events^32^ and gastrointestinal perforation in TOF treatment^33^. Thus, in JAKi treatment, aging may not affect efficacy but may attenuate safety.

With regard to sex, females had higher risk of HZ compared with males in TOF treatment^30^. HZ is one of the most frequent adverse events in JAKi treatment, and has higher incidence in Japan, compared with western countries^34^. This may lead to the higher rate of discontinuation due to toxic adverse events in our present study. RA disease activity tend to be higher in female, whereas clinical response to csDMARDs and bDMARDs appears to be better in male^35^. In our present study, although the baseline disease activities (DAS28-ESR and CDAI) were similar (data not shown), drug retention of JAKi due to lack of effectiveness tended to be higher in male compared to female. This tendency may be similar among these anti-rheumatic agents.

Concomitant use of GC with TOF did not affect clinical or radiographic efficacy^36^. However, the occurrence of HZ doubled in oral GC use^30^; oral GC (> 7.5 mg/day of PSL) was a risk factor for serious infections (including HZ) in TOF treatment^31^. In the present study, patients with PSL ≥ 5 mg/day were at higher risk of toxic adverse events, similar to the results for bDMARDs in Japanese RA patients^19,37^.

Another concern is whether the number or mode of action of prior bDMARDs or JAKi may affect the drug retention of JAKi. Although improvement in disease activity was greatest in the bDMARDs-naïve group, both BAR and TOF were effective in patients refractory to multiple bDMARDs^38^. In addition, prior use of bDMARDs did not affect the clinical efficacy of BAR^39^; the clinical efficacy of BAR was similar regardless of previous multiple bDMARD use^40^. Concerning the mode of action of prior bDMARDs or JAKi, prior use of non-TNFi (n = 31, including aIL-6R and CTLA4-Ig) or JAKi was associated with diminished improvement of DAS28-C-reactive protein (CRP) in BAR treatment^40^. However, non-TNFi (such as aIL-6R and JAKi) may overly downregulate CRP levels by inhibiting IL-6 signalling regardless of its actual disease activity. Therefore, using DAS28-CRP to evaluate disease activity in aIL-6R or JAKi treatment may overestimate their clinical response, and also underestimate following treatment response. Considering the underlying mechanisms, BAR inhibits JAK1 and JAK2 signalling, while TOF inhibits JAK1 and JAK3 signalling, which are mainly involved in IL-6 production^2^. Thus, patients who showed ineffectiveness to aIL-6R may not be fully rescued by BAR or TOF. However, JAK2 is also involved in Granulocyte Macrophage Colony-Stimulating Factor (GM-CSF), which initiates arthritis and pain^41^, and interferon-γ production, which activates macrophages. JAK3 is also involved in IL-2 and IL-21 production, which promote T-cell activation and differentiation, and play important roles in the pathology of RA^42^. Thus, some patients who are dominated by cytokines other than IL-6 may be rescued by switching between BAR and TOF, although further detailed examinations are required.

MTX inhibits not only IL-6 but also inhibits IL-1, matrix metalloproteinases and RF, which play important roles in joint destruction^43^. Indeed, BAR monotherapy was inferior to BAR + MTX in radiographic progression^44^. However, drug retention of BAR due to ineffectiveness^45^ and also drug retention of BAR and TOF in our present study were not significantly affected by concomitant MTX. Taken together, the effectiveness of JAKi in inhibiting joint destruction may be superior in combination with MTX, although drug retention based on clinical settings may be similar compared with monotherapy. The effectiveness of low-dose MTX in Japanese populations should be considered. Intra-erythrocyte MTX-polyglutamate concentration, which is considered a useful biomarker of MTX efficacy, was 65 nmol/L with MTX of 13.4 mg/week in patients from the United States, and reached 94 nmol/L with MTX of 10.3 mg/week in Japanese patients^46^.

The limitations of the present study are as follows. First, although patients were followed by experienced senior rheumatologist of university-related hospitals, the reasons for discontinuation depended on the decisions of different physicians without standardized criteria. Second, according to the Japanese guidelines, TNFi, aIL-6R, or CTLA4-Ig are equally recommended in patients who showed inadequate response to csDMARDs, and JAKi are mainly recommended in patients who showed inadequate response to bDMARDs, which may differ from that of western countries and also affected the results. Third, as the initial dose of each agent was determined according to the manufacturer’s recommendations, minor changes of dose of each agent during the period couldn’t be monitored. Fourth, comorbidities, which can potentially affect drug retention, were not evaluated. Fifth, a relatively small number of prior JAKi-experienced patients may have affected the results. Sixths, the U.S. Food and Drug Administration recently alerted that increased risk of serious cardiovascular events and malignancy of JAKi compared to TNFi, which may affect the results of long-term treatment. However, the strength of this study is that it is the first to evaluate factors affecting plural JAKi retention, by adjusting clinical backgrounds according to prior history of TNFi, aIL-6R, CTLA4-Ig and JAKi, especially in “difficult-to-treat” RA patients who may not be included in randomized controlled trials.

In conclusion, concerning BAR or TOF treatment, prior history of aIL-6R discontinuation due to ineffectiveness may increase the risk of treatment discontinuation due to ineffectiveness. On the other hand, aging (≥ 75 years), concomitant PSL ≥ 5 mg/day, and female sex may increase the risk of treatment discontinuation due to toxic adverse events. These novel findings may provide new insight for the management of JAKi in clinical practice.

## Data Availability

The datasets used and/or analyzed in the current study are available from the corresponding author on reasonable request.
